# Optimal Production and Biochemical Properties of a Lipase from *Candida albicans*

**DOI:** 10.3390/ijms12107216

**Published:** 2011-10-24

**Authors:** Dongming Lan, Shulin Hou, Ning Yang, Chris Whiteley, Bo Yang, Yonghua Wang

**Affiliations:** 1 School of Chemistry and Chemical Engineering, South China University of Technology, Guangzhou 510640, China; E-Mail: landongming@yanhoo.com.cn; 2 School of Bioscience and Bioengineering, South China University of Technology, Guangzhou 510006, China; E-Mail: wshyn091011@gmail.com (N.Y.); 3 Department of Biochemistry, Microbiology & Biotechnology, Rhodes University, Grahamstown 6139, South Africa; E-Mail: c.whiteley@ru.ac.za; 4 Key Lab of Fermentation and Enzyme Engineering, College of Light Industry and Food Sciences, South China University of Technology, Guangzhou 510641, China

**Keywords:** *Candida albicans* lipases, response surface methodology, biochemical characterization, diacylglycerol, coconut oil

## Abstract

Lipases from microorganisms have multi-faceted properties and play an important role in ever-growing modern biotechnology and, consequently, it is of great significance to develop new ones. In the present work, a lipase gene from *Candida albicans* (CaLIP10) was cloned and two non-unusual CUG serine codons were mutated into universal codons, and its expression in *Pichia pastoris* performed optimally, as shown by response surface methodology. Optimal conditions were: initial pH of culture 6.86, temperature 25.53 °C, 3.48% of glucose and 1.32% of yeast extract. The corresponding maximal lipolytic activity of CaLIP10 was 8.06 U/mL. The purified CaLIP10 showed maximal activity at pH 8.0 and 25 °C, and a good resistance to non-ionic surfactants and polar organic solvent was noticed. CaLIP10 could effectively hydrolyze coconut oil, but exhibited no obvious preference to the fatty acids with different carbon length, and diacylglycerol was accumulated in the reaction products, suggesting that CaLIP10 is a potential lipase for the oil industry.

## 1. Introduction

Lipases, which have large biocatalytic potential in both aqueous and non-aqueous media, find their widespread applications in biomedical sciences and chemical synthesis. It has been proved that most of the lipases from microorganisms have multi-faceted properties and play an important role in ever-growing modern biotechnology [1,2]. Lipases have a special catalytic mechanism which is so-called interfacial activation. Lipases may exist in two different conformations, an open and active conformation and a closed and inactive one. In an aqueous homogeneous media, these forms are in equilibrium shifted towards the closed form. In the presence of hydrophobic surfaces (e.g., a drop of oil), the lipase is adsorbed, shifting the equilibrium towards the open conformation one [3]. However, the two open lipase molecules have a trend to give biomolecular aggregated by interacting with each other via the large hydrophobic surface surrounding the active center of lipases [4,5], which may alter the properties of the lipases and reduce their activity. In contrast, the mechanism “interfacial activation” of lipases could be a new tool for purification, immobilization and modulation of lipases properties [6,7].

Lipases from Candida species such as *Candida rugosa*, *Candida Antarctica* have been intensively studied and widely used in many applications for producing valuable esters [8,9]. Although ten lipases (Lip1-Lip10) have been found in yeast *Candida albicans* (*C. albicans*) [10], the characterization and potential application of most of the lipases has not been reported yet. Until now, only lipase 4 and lipase 5 from *C. albicans* have been biochemically characterized [11,12]. Lipase 4 has been produced in *Saccharomyces cerevisiae* and revealed a very efficient acyltransfer activity [11]. And lipase 5 was active at low temperature and behaved as a cold-active lipase [12].

*C. albicans* obeys a special protein translation system which is different from that in the universal host. *C. albicans* encodes a unique seryl-tRNA (CAG) that should decode the leucine codon CUG as serine [13]. Besides *C. albicans*, several species of yeast in the *Candida* genus, such as *parapsilosus*, *zeylanoides*, *ragosa*, and *melibiosica*, also use CUG as a codon for serine instead of leucine [14]. The methylotrophic yeast *P. pastoris* is considered as a valuable host for the production of various proteins of interest, such as enzyme and vaccine [15]. However, functional productions of lipase from *Candida* genus in *P. pastoris* are unfeasible due to the non-universal codon CUG [12,16]. To overcome this problem, mutagenesis of the unusual CUG serine codon into a universal one by site-directed mutagenesis needs to be conducted before expression in the universal host.

One of the most important applications of lipase is to produce fatty acids or special glycerides from oils [17,18]. For example, full hydrolysis of fish oils to produce poly-unsaturated fatty acids has attracted much attention recently [19]. However, partial hydrolysis of triglycerides also leads to production of valuable glycerides [20]. Another application of lipase is used as an additive in detergents, but the activity of lipase might be inhibited by other components in detergents such as proteases and surfactants. Immobilization of lipase on a suitable support provides its reuse and protects it from protease attack and surfactants inhibition. Immobilization can help to enable the application of enzymes in different solvents, at extremes of pH and temperature [21]. The substrate-specificity, enantioselectivity and reactivity of lipase can also be modified [21].

An effective production of lipases is an essential step in determining their biochemical properties, catalytic behavior and sequence-structure-function relationships. However, it is difficult to isolate the individual isoenzymes from *C. albicans* directly because of their high similarities in physical properties. A feasible approach is to produce a lipase by the recombinant DNA technology combined with manipulating culture conditions in a heterologous expression system. A number of statistical experimental designs, such as central composite design (CCD), with response surface methodology (RSM) have been employed for optimizing enzyme production from microorganisms [22–26]. In contrast to “one-at-a-time” technique, the response surface methodology (RSM), which includes factorial design and regression analysis, helps in evaluating the effective factors and building models to study interaction and select optimum conditions of variables for a desirable response [27].

In the present work, our attempt was to produce lipase 10 from *C. albicans* (CaLIP10) and determine its biochemical properties. The two non-universal serine codons (CTG) in the gene were mutated into universal TCT serine codons by overlap extension PCR. The screened CaLIP10 with high productivity was constitutively expressed in *Pichia pastoris* (*P. pastoris*), and its culture process was optimized by response surface methodology. The recombinant CaLIP10 was purified and characterized by monitoring the effect of pH, temperature, ions, detergents, water-miscible solvents on lipase activity and studying the hydrolysis of coconut oil.

## 2. Results and Discussion

### 2.1. Gene Mutation by an Overlap Extension PCR

**Figure 1:**
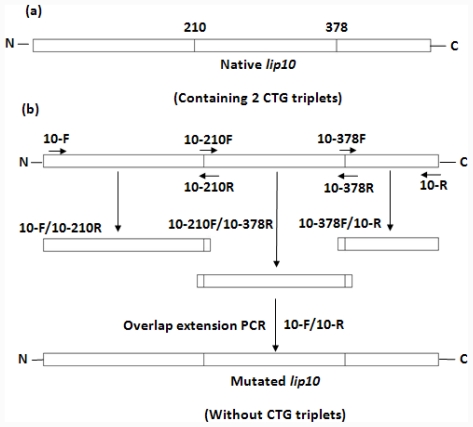
Scheme of the simultaneous multiple mutagenesis introduced by overlap extension PCR for the replacement of the two non-universal CTG-serine codons (amino acid position 210 and 378) in CaLIP10 for universal TCT-serine codons. (**a**) Two CTG-serine residues are indicated with their residue numbers along the polypeptide chain of *lip10*; (**b**) The arrows indicate the mutagenic primers which are used to alert the two CTG triplets in *lip10* gene.

There were two non-universal CUG-Ser codons (residue number 210 and 378) in the *C. albicans lip10* gene (Figure 1a). Both the CUG codons were converted into universal serine codons TCT by using overlap extension PCR (Figure 1b) with the primers in Table 1. The mutated *lip10* DNA sequence, termed as *lip10-dm*, was confirmed by full-length DNA sequencing.

### 2.2. Screening Strains with High Yield of CalIP10

**Figure 2:**
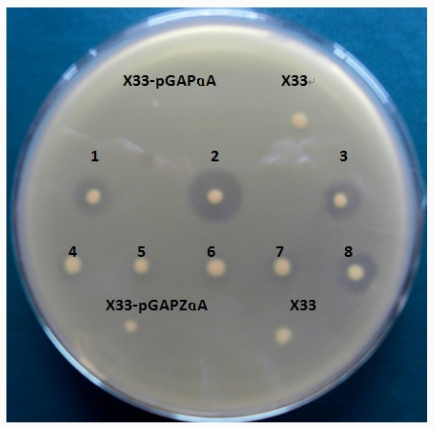
Screening the recombinant transformants on tributyrin-emulsion YPD plates. Number 1–3, 8: transformants harboring mutated *lip10* gene; Number 4–7: transformants harboring wild type genes. X-33 and X-33 harboring empty expression vector pGAP-ZαA were used as a control.

*P. pastoris* transformants containing *lip10* and *lip10-dm* were screened using 1% tributyrin-emulsion YPD plates. The clear zone on the opaque tributyrin emulsion identified the lipase-secreting transformants. It can be seen from Figure 2 that a clear zone was formed around wild type *lip10* and mutated *lip10-dm*, and the diameter of the transparent ring formed by the mutant was larger than that formed by the wild type. Transformants, which contained wild lip10 and mutant lip10-dm, were cultured in YPD medium to investigate the enzymatic activity. After 72 h, the lipase activities were 2.05 U/mL, and 4.92 U/mL, respectively. These results indicated that both residue Ser-210 and Ser-378 played an important role in lipolytic activity. The reason may be that Ser-210 and Ser-378 are key amino acids which are involved in the active domain, or they have impact effect on the correct folding of protein. Roustan *et al*. reported that the mutagenesis of CUG codons in one of the lipases from *C. albicans* (CaLIP4) resulted in a ten-fold lipase activity increase [11].

### 2.3. Optimization of Culture Conditions for the Production of CaLIP10

The CCD was predicted to obtain the possible culture conditions for CaLIP10 production from recombinant *P. pastoris*. Experiments 1–30 were performed with different combinations and those from 10 to 15 were under the same conditions (Table 2). The actual lipase activity obtained in the experiments and predicted lipase activity produced by the model are also given in Table 2. By analysis of the experimental data, the quadratic equation model was as follows:

(1) $$\begin{array}{l} \text{y}=-56.37102+2.28687x_{1}+6.23155x_{2}+8.41193x_{3}+3.57600x_{4}-0.031967x_{1}x_{2}\\ -0.10758x_{1}x_{3}-0.066154x_{1}x_{4}-0.027399x_{2}x_{3}+0.15677x_{2}x_{4}+0.083731x_{3}x_{4}\\ -0.034388x_{1}^{2}-0.39273x_{2}^{2}-1.73641x_{3}^{2}-0.45131x_{4}^{2} \end{array}$$

where *y* is the predicted response; *x*_1_, *x*_2_, *x*_3_ and *x*_4_ are the uncoded values: Temperature, initial pH of culture, concentration of yeast extract content and concentration of glucose. The analysis of variables for the model and factors are shown in Table 3 which lists the regression coefficients calculated by the model for *β*_i_, *β*_ii_, *β*_ij_ along with significance levels of the terms. From *p*-values of terms, it can be seen that the linear term of temperature, yeast extract concentration and glucose concentration as well as the square term for temperature, pH and glucose concentration, were highly significant on CaLIP10 activity; the linear term of initial pH (0.0126) was the least significant. The interactions of initial pH and glucose concentration (0.0014), temperature and glucose concentration (0.0009) have a minor effect on the CaLIP10 activity while the interaction of initial pH and yeast extract (0.8225) has the most significance on the activity of CaLIP10.

The coefficients of correlation and variation and *F*-value are given in Table 4. The mathematical model was very reliable with an *R*^2^ value of 0.9814. The closer the *R*^2^ is to 1, the better the model fits the experimental data, the less the difference between the predicted and the observed values. The compared *F*-value of 56.64214 was greater than the *F* (14, 15) value in the statistic table at 1% level. It reflected the significance of the model. In summary, those values indicated a satisfactory fitness of the quadratic model and were adequate to represent the actual relationship between the responses (lipolytic activity) and the significant variables.

**Figure 3:**
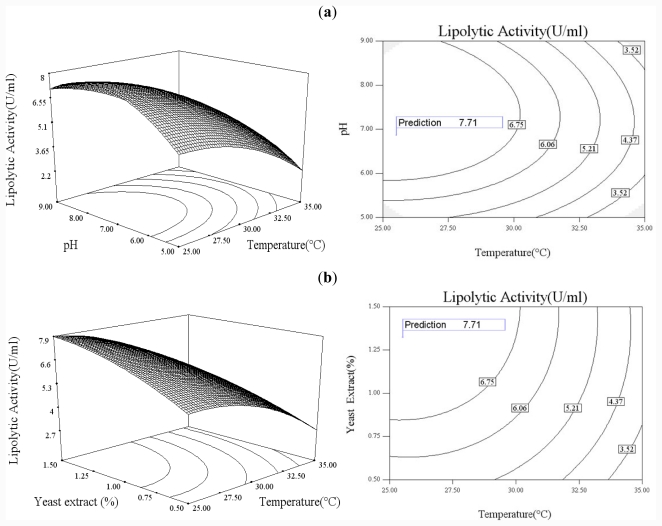

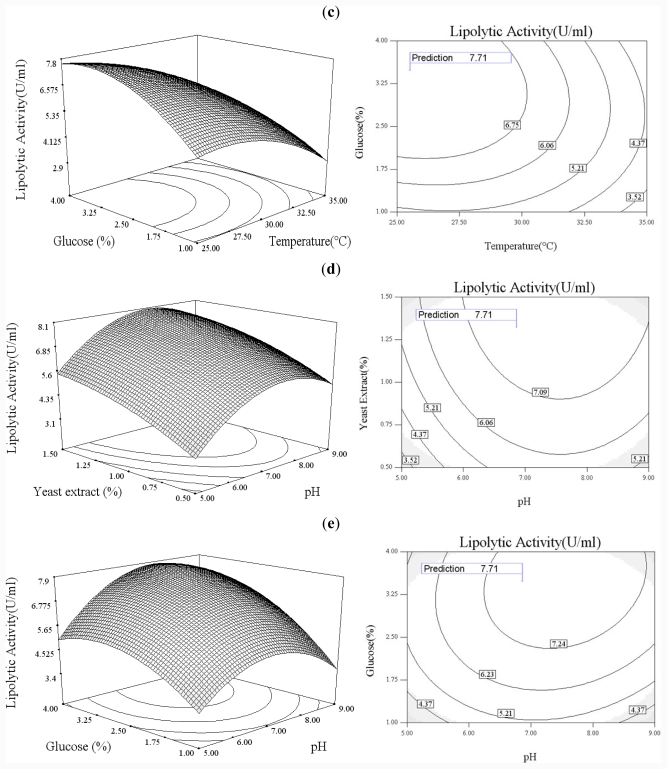

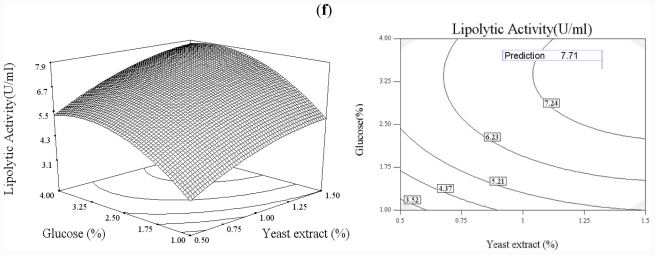
Response surface plots and planned series of contour plots of four independent variables and their effects on responses (lipolytic activity): (**a**) The effects of temperature, pH, and their mutual interaction on lipolytic activity with constant level of concentration of yeast extract and glucose (1.32% and 3.48%, respectively); (**b**) The effects of temperature, yeast extract concentration, and their mutual interaction on lipolytic activity with constant level of glucose concentration and initial pH (3.48% and 6.86, respectively); (**c**) The effects of temperature, glucose concentration, and their mutual interaction on lipolytic activity with constant level of yeast extract concentration and initial pH (1.32% and 6.86, respectively); (**d**) The effects of yeast extract concentration and pH, and their mutual interaction on lipolytic activity with constant level of temperature and glucose concentration (25.53 °C and 3.48%); (**e**) The effects of glucose concentration and pH, and their mutual interaction on lipolytic activity with constant level of temperature and yeast extract concentration (25.53 °C and 1.32%, respectively); (**f**) The effects of glucose and yeast extract concentration, and their mutual interaction on lipolytic activity with constant level of temperature and initial pH (25.53 °C and 6.86, respectively).

The three-dimensional response surface and contour curves for the four variables are shown in (Figure 3). The response surface representing CaLIP10 activity was a function of two tested variables with the other two variables being maintained at their optimal level. It was clear and obvious to understand the interaction between two factors and to predict their optimal levels.

From the response surface, it indicated that the optimal initial pH of the culture was 6.86 for CaLIP10 activity (Figure 3a,d,e) since a lower or higher initial pH decreased not only the activity of CaLIP10 but had a negative effect on the cell growth. The corresponding cell density (OD_600_) was 13.5, 16.8 and 26 when the initial pH of the culture medium was selected to be 3, 11 and 7 respectively with other process parameters being the same. The reason why recombinant CaLIP10 production was related to the cell growth was that the constitutive promoter was used in the vector system. Culture temperature made an impact on the CaLIP10 production (Figure 3a–c) with temperatures over 30 °C being detrimental to the cell growth (data not shown) and protein production while a lower temperature (25.53 °C) favored maximal lipase production, probably because the higher specific growth rates caused much more metabolic burden to be placed on the cell. Yeast extract concentration of 1.32% and a glucose concentration around 3.48% gave the maximal predicted lipase production (Figure 3b–f). Yeast extract was an important nitrogen source for yeast growth and any concentration of yeast extract of less than 1.32% could not fully support the cell growth [28]. However, it was reported that yeast extract rich in amino acids and peptides displayed a repressive effect on enzyme production when used at a higher concentration (>1.5%, m/v) [29]. A high glucose concentration (>10%, m/v) might be unfavorable for enzyme production due to the osmotic pressure or the carbon catabolite repression [30,31]. No significant repression effect of glucose on CaLIP10 production was found at a concentration of less than 5.5% in our study.

The above observations indicated that the maximum production of CaLIP10 (7.89 U/mL) was obtained when the initial pH of the culture, the temperature, the yeast extract concentration and the glucose concentration were at about 6.86, 25.53 °C, 1.32% and 3.48% respectively. In order to verify the predicted results, the experiment was performed under the optimized culture condition and the experimental value was 8.06 U/mL, indicating that experimental and predicted values of CaLIP10 yield were in a good agreement.

### 2.4. Purification of Recombinant CaLIP10

**Figure 4:**
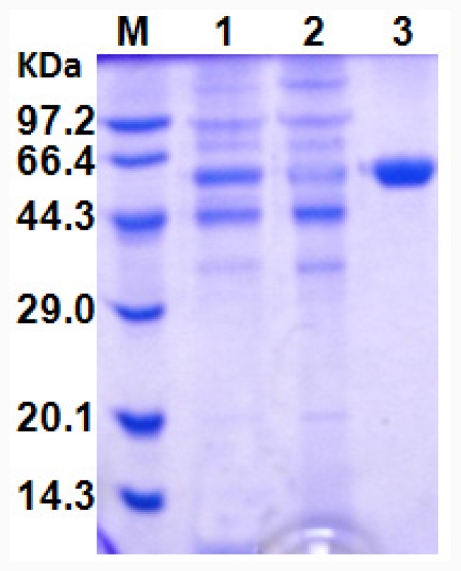
SDS-PAGE analysis for the purification of CaLIP10: lane M, molecular mass standards indicated in kDa; lane 1, supernatant of culture medium; lane 2, sample eluted with equilibrated buffer (sodium acetate buffer, pH 5.5); lane 3, purified CaLIP10.

The recombinant CaLIP10 was efficiently purified by cation-exchange chromatography, and its molecular mass was determined to be about 50 kDa by SDS-PAGE analysis (Figure 4). After purification, the specific activity of CaLIP10 increased 5.68 fold with a recovery of 86.01% (Table 5). This pure CaLIP10 was used for further analysis of biochemical properties.

### 2.5. Analysis of the Biochemical Properties of Recombinant CaLIP10

#### 2.5.1. pH and Temperature

**Figure 5:**
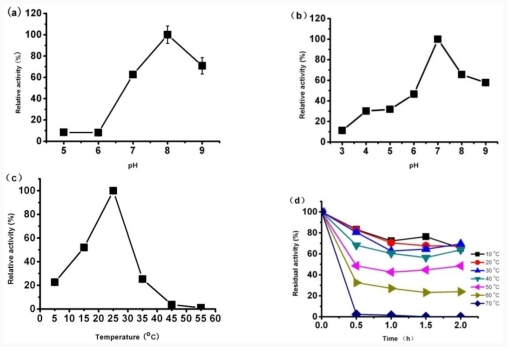
Effects of pH and temperature on lipolytic activity and stability of purified CaLIP10. Activities are shown as percentages of the maximum activity (64.51 U/mg). All experiments were conducted in triplicate. Values are means ± SD from three independent experiments. (**a**) pH effect on the activity of CaLIP10 was measured at various pHs from 4.0 to 9.0 at 25 °C; (**b**) pH effect on stability of CaLIP10. The enzyme was investigated after incubation in a range of pH (3.0–9.0) for 20 h; (**c**) Temperature effect on lipolytic activity of CaLIP10 was measured at various temperatures from 5–55 °C; (**d**) Temperature effect on stability of CaLIP10 was investigated after incubation at various temperatures from 10–70 °C for 2 h at pH 8.0.

The effect of pH (Figure 5a,b) and temperature (Figure 5c,d) on the lipase activity and stability of CaLIP10 were determined spectrophotometrically using *p*-nitrophenol caprylate (*p*-NPc) as substrate. The recombinant CaLIP10 was found to be most active at pH 8.0 and exhibited very low activity (<10%) at pH 5–6 (Figure 5a). The similar results were observed with lipases from *Pseudomonas fluorescens* JCM5963 and *Bacillus coagulans* BTS-3 [32,33]. Stability was tested after incubation at different pH value buffer for 24 h. The activity of CaLIP10 decreased by more than 50% after incubation at buffer with the pH below 6 for 24 h, while it was relatively stable in an alkaline environment (Figure 5b). Different pH could affect the ionization state of the amino acids which dictate the primary and secondary structure of the enzyme and hence, controls its overall activity.

The optimum temperature for CaLIP10 activity was 25 °C, but the relative activity maintained about 20% of maximal CaLIP10 activity when the temperature was at 5 °C (Figure 5c) supporting Margesin’s definition [34] that CaLIP10 might not be identified as a cold-adapted lipase. The thermo stability of recombinant CaLIP10 was investigated by incubation at different temperature for 2 h at pH 8.0. The relative activity of recombinant CaLIP10 was greater than 60% after incubation of CaLIP10 at temperature below 40 °C for 2 h, while its activity decreased dramatically after the incubation temperature above 50 °C (Figure 5d).

In the optimization of the CaLIP10 production section, low enzyme activity was observed in pH 3.0. One reason might be that the growth of cell was affected under low pH value, which might decrease the production of recombinant enzyme. Meanwhile, the configuration of enzyme might be destroyed at low pH value, which resulted in decreasing the activity of enzyme. The enzyme was more stable at low temperature (Figure 5d). This result was consistent with the fact that maximal lipase production was achieved at a lower cultural temperature (25.53 °C). Therefore, the recombinant yeast should be cultured at relative lower temperature to maintain the activity of CaLIP10.

#### 2.5.2. Effect of Chemicals, Inhibitors, Detergents and Water-Miscible Solvents

The effects of various chemicals, detergents/inhibitors and water-miscible solvents on lipolytic activity of CaLIP10 are shown in Table 6. Lipase activity decreased significantly as the concentration of Zn^2+^, Cu^2+^, Mg^2+^, Fe^3+^, Ca^2+^, Mn^2+^, Hg^2+^, Li^+^, Ni^2+^, Ba^2+^ and Co^2+^ increased. PMSF, which is a serine inhibitor, decreased activity of CaLIP10 distinctly when its concentration increased from 1 to 5 mM. The addition of 1 mM and 5 mM of EDTA did not significantly affect the enzyme activity, indicating that CaLIP10 was not a metalloenzyme.

Different surfactants have a different influence on the CaLIP10 activity. Relative activities of CaLIP10 were 78.87, 98.48 and 102.48% when 1% of Triton X-100 (HLB-13.0), Tween-80 (HLB-15.0), and Tween-20 (HLB-16.7) were used respectively, suggesting that CaLIP10 activity increased with the increment of hydrophilic-lipophilic balance (HLB) value. The results indicated that the hydrophilicity of non-ionic surfactants might help to increase CaLIP10 stability. However, a higher concentration (5%) of Tween-80 and Tween-20 showed a detrimental effect on CaLIP10 activity, which may be due to the fact that the excessive adsorption of the surfactants on the enzyme surface results in a diffusional limitation on the reaction [35]. In contrast to the above results, SDS placed a drastic inhibitory effect on activity of CaLIP10 possibly because of its negative charge and its denaturing capability.

The stability or enhancement of enzyme activity in the presence of organic solvents was generally considered as a valid feature, as it was a prerequisite for the synthesis of valuable compounds in non-aqueous solvents. It could be seen from Table 6 that CaLIP10 activity decreased after being treated with different solvents, but no obvious relation was found between CaLIP10 activity and the Log *P* of organic solvents. It was reported that the hydrophilic solvents may strip off the water layer from the surface of the enzyme and compete strongly for hydrogen bonds between protein atoms, leading to protein unfolding and subsequent denaturation [36]. Though CaLIP10 activity dramatically decreased after being treated with acetone, methanol and isopropanol, it still retained more than 73% of maximal activity when CaLIP10 was treated with ethanol for 1 h.

### 2.6. Hydrolysis of Coconut Oil

**Figure 6:**
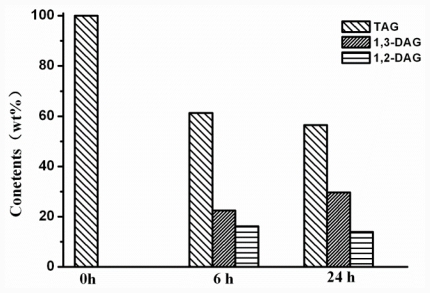
Hydrolysis of coconut oil by CaLIP10. The content of TAG, 1,3-DAG and 1,2-DAG were analyzed after hydrolysis at 6 h and 24 h.

To investigate the hydrolysis ability toward oil, the coconut oil was used as a substrate. As shown in Figure 6, 38.64% and 43.56% of Triglyceride (TAG) were converted after 6 h and 24 h of reaction. It was found that the hydrolysis reaction almost did not progress from 6 to 24 h. The pH of the reaction solution might decrease due to accumulation of the released free fat acid, which led to decrease the activity of CaLIP10 since it was low activity and stability at low pH value. A buffering system will be used to investigate the hydrolysis reaction of the coconut oil in a subsequent study. Diacylglycerol (DAG) products were accumulated with the reaction proceeded, and 1,3-DAG was the predominant component in the DAG mixture. We are not sure whether CaLIP10 really is specific for position 2 based on the existent results. Lipase with *sn-2* preference has great potential applications in industries. Until now, lipase A from *Candida Antarctica* (CALA) has been reported to be the only known lipase with a *sn2*-preference towards triglycerides [9]. Therefore, we will make effort to ensure this point in further studies. The composition of released fatty acids were analyzed (Table 7), and the CaLIP10 showed no obvious preference to the fatty acids with different carbon length.

## 3. Experimental Section

### 3.1. Materials

All chemicals used in the study were analytical pure grade. The substrates *p*-nitrophenol caprylate (*p*-NPc) were purchased from Sigma-Aldrich (Germany). Other reagents were purchased from Takara (Japan) or Omega (USA).

### 3.2. Strains, Plasmids and Culture Media

Strain *C. albicans* ATCC 10231 was purchased from Guangdong Microbial Culture Collection Center (China). *P. pastoris* X33 and *pGAPZαA* (Invitrogen) were used as host and vector, respectively, for heterologous expression of the lipase, while *E. coli* DH5α and plasmid pBluescript SK vector (BSK, Stratagene) were used for vector construction. *P. pastoris* X33 was grown in shake-flasks at 30 °C in YPD medium (per liter: 10 g yeast extract, 20 g peptone, 20 g glucose). YPD containing 100 μg/mL Zeocin (Invitrogen) was used for screening of transformants. *E. coli* strains containing recombinant plasmids were cultured in Luria Bertani (LB) medium at 37 °C and supplemented with Zeocin when needed.

### 3.3. Cloning and Mutagenesis of CalIP10

*C. albicans* genomic DNA was prepared using a fungal genome extraction kit (Omega) according to the manufacturer’s instruction. The mature protein-coding sequence without the signal peptide of *C. albicans* lipase 10 (namely *lip10*) was amplified by polymerase chain reaction (PCR) from genomic DNA. Specific primers 10F/10R (Table 1) were used. The PCR product was first purified and digested by restriction enzymes, then ligated with predigested pBluescript SK vector, and was termed pBluescript-*lip10*. The plasmid construct was used as the template for the mutagenesis to convert 2 non-universal CTG-serine codons (residue number 210 and 378) in CaLIP10 into universal TCT-serine codons. The simultaneous multiple mutagenesis was carried out by the overlap extension PCR [37]. The product of the mutagenesis was ligated into the pBluescript SK vector, and was termed pBluescript-*lip10*-*dm*. The sequence of mutated *lip10* containing the mentioned replacements was confirmed by the full-length DNA sequencing. The scheme for mutation is shown in Figure 1 and the mutagenic primers are shown in Table 1. Subcloning of *lip10* and *lip10-dm* into the expression vector *pGAPZαA* were performed to generate pGAPZαA-*lip10* and pGAPZαA-*lip10*-*dm*.

### 3.4. Screening Recombinant *P. pastoris* with High Yield of CaLIP10

The plasmid pGAPZαA-*lip10* and pGAPZαA-*lip10*-*dm* were linearized with *Avr*II (NEB, USA) and transformed into *P. pastoris* X33 by electroporation using Gene Pulser (Bio-Rad) apparatus. Transformants were screened on YPD plates (containing 2% agar and 100 μg/mL Zeocin) to isolate Zeocin-resistant clones. Individual colonies were pitched and patched on 1% tributyrin-emulsion YPD plates to identify lipase-secreting transformants which would form a clear zone on the opaque tributyrin emulsion. *P. pastoris* transformed with pGAPZαA was used as a negative control. Strains with high yield of CaLIP10 were maintained at −80 °C in 50% glycerol (v/v).

### 3.5. Optimizing the Production of CaLIP10 by Central Composite Design

#### 3.5.1. Culture of the Recombinant *P. pastoris*

Seed medium consisting of YPD was inoculated and incubated in a rotary shaker (30 °C, 250 rpm, 24 h) and then 5 mL was used to inoculate 50 mL of production medium (YPD) in a 300 mL glass flask. This culture was grown under the different conditions to optimize the culture process.

#### 3.5.2. Experimental Design

In order to explore the effect of culture medium (glucose, yeast extract) and growth condition (pH and temperature), a statistical approach using the Box–Wilson central composite design (CCD) was conducted [38]. In this study, the four independence variables were studied at five levels (−2, −1, 0, 1, 2) and a 4-factor-5-level CCD that consisted of 30-run experiments (Table 2) generated by Design Expert 7.0 (Stat-Ease Inc., USA) was used. The lipolytic activity of lipase was taken as responses.

The CCD experimental results were fitted with a second-order polynomial equation of Equation 2 [39]:

(2) $$Y=\beta_{k0}+\sum_{i=1}^{4}\beta_{ki}x_{i}+\sum_{i=1}^{4}\beta_{kii}x_{i}^{2}+\sum_{i=1}^{3}\sum_{j=i+1}^{4}\beta_{kij}x_{i}x_{j}$$

where *Y* is response (lipolytic activity); *β_k_*_0_, *β_i_*, *β_kii_*, and *β_kij_* are constant coefficients and *x_i_* the uncoded independent variable. The analysis of variance (ANOVA) was performed to evaluate significance of the model and coefficients.

### 3.6. Purification of Recombinant CaLIP10

All procedures were performed at 4 °C. The culture broth was centrifuged (12,000 × *g*, 4 min) to remove the cells. The crude enzyme solution was adjusted to pH 5.5 using HCl, and applied onto a CM Sepharose^TM^ Fast Flow column (CM FF, 1.6 cm × 28 cm, GE Healthcare) equilibrated with 20 mM sodium acetate buffer (pH 5.5). The column was washed with 20 mM sodium acetate buffer (pH 5.5) and then bound protein was eluted with a gradient of the same buffer containing 0–1.0 M NaCl at a flow rate of 3 mL/min. The active fractions (50 mL) were pooled, concentrated and 10 mL loaded onto the Sephadex G25 column (2.5 cm × 20 cm) equilibrated with 100 mM sodium phosphate buffer (pH 7.0). The column was then washed with the same buffer and the lipase-active fractions pooled and stored at 4 °C.

The molecular mass of the purified recombinant lipase was determined in denaturing conditions by SDS-PAGE with 5% polyacrylamide-stacking gel and a 12% polyacrylamide-resolving gel. The total protein in the samples was quantified with a BCA Protein Assay Kit (Sangon, Shanghai, China) using bovine serum albumin as standard according to the manufacturer’s instruction.

### 3.7. Lipase Activity Assay

The lipase activity was determined spectrophotometrically using *p*-nitrophenol caprylate as substrate. The reaction mixture consisted of 50 mM Tris-HCl buffer (pH 8.0), 2 mM *p*-NPc and an appropriate amount of the lipase and the amount of released *p*-nitrophenol quantified after 5 min reaction time by its absorbance at 405 nm. One unit of activity was defined as the amount of enzyme needed to release 1 μmol of *p*-nitrophenol per minute under the assay conditions.

### 3.8. Characterization of CaLIP10

#### 3.8.1. Effect of Temperature on CaLIP10 Activity and Stability

Effect of temperature on the lipase activity was determined by measuring the hydrolytic activity at different temperatures in 50 mM Tris-HCl buffer (pH 8.0). The temperature was set as 5 °C, 15 °C, 25 °C, 35 °C, 45 °C and 55 °C. The activity was expressed as percent relative activity in relation to the temperature optimum, which was considered as 100%.

The thermostability was investigated by incubating the lipase at the different temperatures for 2 h and measuring its activity every 30 min. The activity was expressed as percent relative activity with respect to maximum activity, which was considered as 100%.

#### 3.8.2. Effect of pH on the CaLIP10 Activity and Stability

The optimum pH was investigated by measuring the hydrolytic activity of lipase at 25 °C at different pH between 3.0 and 9.0 by using the following buffers: 100 mM sodium acetate buffer (pH 3.0–5.0), 100 mM sodium phosphate buffer (pH 6.0, pH 7.0), 50 mM Tris-HCl buffer (pH 8.0) and 50 mM Gly-NaOH buffer (pH 9.0). The activity was expressed as percent relative activity with respect to maximum activity, which was considered as 100%.

To analyze pH stability of the lipase, the purified CaLIP10 was pre-incubated (4 °C, 20 h) in buffers with each of the different pH value and the residual activity was measured The activity was expressed as percent relative activity with respect to maximum activity, which was considered as 100%.

#### 3.8.3. Effect of Metal Ions, Inhibitors, Detergents and Water-Miscible Solvents on the CaLIP10 Activity

The influence of various chemicals, inhibitors, surfactants and water-miscible solvent on the hydrolytic activity was determined by detecting the residual activity at 25 °C after incubating (4 °C, 1 h) the pure CaLIP10 in 50 mM Tris-HCl buffer (pH 8.0) containing each of various metal ions (1 mM and 5 mM), each of detergents/inhibitors (0.1% v/v) or each of water-miscible solvents (30% v/v). The metal ions included ZnSO_4_, CuSO_4_, MgSO_4_, FeCl_3_, CaCl_2_, MnSO_4_, HgCl_2_, LiCl, NiCl_2_, BaCl_2_, CoCl_2_; inhibitors included ethylenediaminetetraacetic acid (EDTA) and phenylmethylsulfonyl fluoride (PMSF); detergents included Tween 20, Tween 80, Triton X-100 and sodium dodecyl sulphate (SDS and water-miscible solvents included methanol, ethanol, acetone and isopropanol. The percentage residual activities were expressed in comparison with a standard assay mixture with no metal ions, detergents/inhibitors or water-miscible solvents added.

#### 3.8.4. Hydrolysis of Coconut Oil

5 g of coconut oil and 0.5 g of distilled water were added into a 50 mL Florence flask, and the reaction was preceded under magnetic stirring (200 rpm) at 25 °C with an addition of 100 U of CalIP10 per gram of coconut oil. About 0.1 g of the reaction mixture was withdrawn periodically and centrifuged at 10,000 rpm for 5 min. The upper layer was transferred into another tube, and anhydrous sodium sulfate was added to remove the residual water. And then 1 mL of *n*-hexane and 2-propanol (15:1 v/v) was added to the mixture and swirled thoroughly. The supernatant was collected for HPLC analysis by centrifugation at 10,000 rpm for 1 min.

#### 3.8.5. Analysis of Hydrolytic Products by HPLC and GC

After 48 h of hydrolysis of coconut oil, 100 mg of reaction mixture were withdrawn into a separatory funnel, and then hexane (10 mL) and phenolphthalein solution was added. The reaction mixture was neutralized with KOH 0.5 N hydroehanolic solution (30% ethanol). The lower phase was acidified (pH 1.0) with 6 N HCl. The liberated free fatty acids were then extracted with 50 mL of hexane. The hexane layer was dried over anhydrous sodium sulfate, and the solvent was removed with a rotary evaporator at 40 °C. Free fatty acids were methylated and analyzed by GC [40]. The dacylglycerols in the reaction mixture were analyzed by HPLC [41].

## 4. Conclusions

Functional expression of CaLIP10 in *P. pastoris* and indicated biochemical properties of CaLIP10 were performed. 210 and 378-CUG-Ser codons play an impact role in the lipase activity, and about 2-fold activity increased when they were mutated into universal TCT serine codons. The lipolytic activity of CaLIP10 was further increased by 64% at optimal conditions under initial pH of culture at 6.86, temperature at 25.53 °C, 3.48% of glucose and 1.32% of yeast extract.

The optimum conditions for lipolytic activity were 25 °C and pH 8. Ions, such as Fe^3+^ at a high concentration (5 mM) reduced lipolytic activity of CaLIP10 dramatically. The hydrophilicity of non-ionic surfactants at low concentration (1%) might help to increase the stability and activity of CaLIP10 to some degree. CaLIP10 exhibited a good stability in non-ionic surfactants and retained more than 73% of maximal activity after being treated with ethanol. CaLIP10 could effectively hydrolyze coconut oil, but exhibited no obvious preference to the fatty acids with different carbon lengths, and diacylglycerol was accumulated in the reaction products, suggesting that CaLIP10 is a potential lipase for application in the oil industry.

## Figures and Tables

**Table 1:** Primers for *lip10* gene cloning and mutation.

Primer	Sequence
10-F	ACGCGTCGAC TCCTTGATAGGATTGACGCCAC
10-R	CGCGGATCCGCGGCGTTAAATATATTTTCTAATATAAGCGAA
10-210R	TCTGGGGCATATTT **AGA** CTGTAAACTTATTGC
10-210F	GCAATAAGTTTACAG **TCT** AAATATGCCCCAGA
10-378R	CTAAATGACCTGC **AGA** CAAATCTTCAGCAAAT
10-378F	ATTTGCTGAAGATTTG **TCT** GCAGGTCATTTAG

**Table 2:** 4-Factor-5-level response surface analysis.

No.	Coded values and actual values				Actual lipolytic activity (U/mL)	Predicted lipolytic activity (U/mL)	Residual
	Temperature (°C) a*x*_1_	pH a*x*_2_	Yeast extract (%, m/v) a*x*_3_	Glucose (%, m/v) a*x*_4_			
1	−2 (20)	0 (7)	0 (1)	0 (2.5)	6.27	5.62	0.65
2	−1 (25)	−1 (5)	−1 (0.5)	1 (4)	2.68	2.79	−0.11
3	−1 (25)	−1 (5)	1 (1.5)	1 (4)	4.60	5.24	−0.64
4	−1 (25)	−1 (5)	1 (1.5)	−1 (1)	3.19	3.51	−0.32
5	−1 (25)	−1 (5)	−1 (0.5)	−1 (1)	1.18	1.31	−0.13
6	−1 (25)	1 (9)	1 (1.5)	−1 (1)	3.37	3.73	−0.36
7	−1 (25)	1 (9)	−1 (0.5)	−1 (1)	1.64	1.64	0.00
8	−1 (25)	1 (9)	−1 (0.5)	1 (4)	4.99	5.00	0.01
9	−1 (25)	1 (9)	1 (1.5)	1 (4)	6.92	7.33	−0.41
10	0 (30)	0 (7)	0 (1)	0 (2.5)	6.31	6.33	−0.02
11	0 (30)	0 (7)	0 (1)	0 (2.5)	6.41	6.33	0.08
12	0 (30)	0 (7)	0 (1)	0 (2.5)	6.55	6.33	0.22
13	0 (30)	0 (7)	0 (1)	0 (2.5)	6.21	6.33	−0.12
14	0 (30)	0 (7)	0 (1)	0 (2.5)	6.20	6.33	−0.13
15	0 (30)	0 (7)	0 (1)	0 (2.5)	6.23	6.33	−0.10
16	0 (30)	0 (7)	0 (1)	−2 (−0.5)	0.81	0.72	0.09
17	0 (30)	0 (7)	0 (1)	2 (5.5)	4.26	3.82	0.44
18	0 (30)	0 (7)	2 (2)	0 (2.5)	7.33	6.32	1.01
19	0 (30)	0 (7)	−2 (0)	0 (2.5)	2.39	2.87	−0.48
20	0 (30)	−2 (3)	0 (1)	0 (2.5)	0.01	−0.52	0.53
21	0 (30)	2 (11)	0 (1)	0 (2.5)	0.62	0.63	−0.01
22	1 (35)	1 (9)	1 (1.5)	1 (4)	2.22	2.43	−0.21
23	1 (35)	1 (9)	1 (1.5)	−1 (1)	0.65	0.81	−0.16
24	1 (35)	1 (9)	−1 (0.5)	1 (4)	1.22	1.17	0.05
25	1 (35)	−1 (5)	1 (1.5)	1 (4)	1.34	1.61	−0.27
26	1 (35)	1 (9)	−1 (0.5)	−1 (1)	0.09	−0.20	0.29
27	1 (35)	−1 (5)	−1 (0.5)	1 (4)	0.26	0.24	0.02
28	1 (35)	−1 (5)	−1 (0.5)	−1 (1)	0.90	0.75	0.15
29	1 (35)	−1 (5)	1 (1.5)	−1 (1)	1.53	1.87	−0.34
30	2 (40)	0 (7)	0 (1)	0 (2.5)	0.05	0.16	−0.11

a Actual variables are given in parentheses.

**Table 3:** Effect estimate for the hydrolytic activity production of CaLIP10 by recombinant *P. pastoris*.

Source	Coefficient Estimate a	Standard Error a	*F*-value	*p*-value (Prob > *F*)
*x*_1_	−1.36582	0.097992	56.64214	<0.0001
*x*_2_	0.277726	0.097992	194.2682	0.0126
*x*_3_	0.864656	0.097992	8.032446	<0.0001
*x*_4_	0.773923	0.097992	77.85784	<0.0001
*x*_1_*x*_2_	−0.31967	0.120016	62.37505	0.0177
*x*_1_*x*_3_	−0.26894	0.120016	7.094752	0.0406
*x*_1_*x*_4_	−0.49615	0.120016	5.021685	0.0009
*x*_2_*x*_3_	−0.0274	0.120016	17.09048	0.8225
*x*_2_*x*_4_	0.470295	0.120016	0.05212	0.0014
*x*_3_*x*_4_	0.062798	0.120016	15.35556	0.6084
*x*_1_^2^	−0.85971	0.091663	0.273792	<0.0001
*x*_2_^2^	−1.57091	0.091663	87.96513	<0.0001
*x*_3_^2^	−0.4341	0.091663	293.7035	0.0003
*x*_4_^2^	−1.01544	0.091663	22.42802	<0.0001

a Coefficient and standard error are termed in coded values.

**Table 4:** Analysis of variance and regression for lipolytic activity of CaLIP10 (quadratic model).

Source	Sum of squares	Degree of freedom	Mean square	*F*-value	*P* > *F*
Model error	182.7526	14	13.05376	56.64214	<0.0001
Residual error	3.456902	15	0.23046		
Total	186.2095	29			
Coefficient of correlation (*R*^2^), 0.9814					
Coefficient of determination (adjusted *R*^2^), 0.9641					
Coefficient of variation(CV), 14.9400%					

**Table 5:** Summary of the Purification of the Recombinant CaLIP10.

Purification step	Total volume (mL)	Enzyme activity (U/mL)	Protein concentration (mg/mL)	Specific activity (U/mg)	Purification factor (fold)	Yield (%)
Culture medium	250	8.06	0.45	17.91	1	100.00
CM FF	55	31.51	0.31	101.65	5.68	86.01

**Table 6:** Effect of various chemicals, detergents and water-miscible solvent on the recombinant lipase activity a.

Chemicals	Relative activity (%)	
	1 mM	5 mM
Zn^2+^	69.13 ± 5.7	40.3 ± 3.16
Cu^2+^	80.29 ± 3.68	56.69 ± 4.09
Mg^2+^	96.56 ± 3.28	66.02 ± 3.87
Fe^3+^	70.06 ± 0.79	47.87 ± 8.16
Ca^2+^	94.20 ± 4.23	80.21 ± 4.41
Mn^2+^	90.74 ± 5.52	71.73 ± 5.66
Hg^2+^	83.89 ± 4.09	58.93 ± 4.00
Li^+^	78.52 ± 10.14	65.30 ± 1.74
Ni^+^	72.38 ± 7.13	51.06 ± 2.38
Ba^2+^	75.40 ± 2.74	59.47 ± 2.21
Co^2+^	88.53 ± 2.4	56.21 ± 1.66
EDTA	70.04 ± 4.23	67.19 ± 1.99
PMSF	86.02 ± 2.2	64.83 ± 2.08

**Detergents**	**Relative activity (%)**	
	**1%**	**5%**

Triton X-100 (13.0) b	78.87 ± 3.52	86.85 ± 2.08
Tween80 (15.0) b	98.48 ± 9.03	79.87 ± 7.13
Tween20 (16.7) b	102.48 ± 7.23	52.18 ± 2.01
SDS (40.0) b	2.00 ± 1.21	1.19 ± 0.16

**Water-miscible solvents**	**Relative activity (%)**	
	**30%(v/v)**	

Methanol (−0.764) c	56.44 ± 4.12	
Ethanol (−0.235) c	73.39 ± 5.57	
Acetone (−0.208) c	59.04 ± 2.22	
Isopropanol (0.074) c	44.14 ± 1.48	

a The purified recombinant lipase was incubated in 0.05 M Tris-HCl (pH8.0) containing each chemicals for 1 h. Residual activities the lipase retained were measured using *p*-NPc as substrate under standard condition. Activities are shown as percentages of the control activity value. Values are means ± SD from three independent experiments;

b The values in parentheses represent the HLB value of detergents;

c The values in parentheses represent the log *P* value of polarity of an organic solvent.

**Table 7:** Composition of fatty acids in coconut oil and released fatty acids after hydrolysis.

Fatty acids	C8:0	C10:0	C12:0	C14:0	C16:0	C18:0	C18:1	C18:2
Composition of fatty acids in the coconut oil	7.44	6.59	49.36	17.48	8.1	6.65	2.96	1.42
Released fatty acids after 48 h of hydrolysis	8.91	6.96	45.67	19.40	8.31	7.96	2.79	–
